# The whole chloroplast genome of *Neomartinella yungshunensis* (Brassicaceae), an unusual wild plant

**DOI:** 10.1080/23802359.2022.2087543

**Published:** 2022-06-22

**Authors:** Weiguo Chai, Huimin Li, Xiang Chen, Jieren Qiu, Pengguo Xia

**Affiliations:** a Institute of Biotechnology, Hangzhou Academy of Agricultural Sciences, Hangzhou, PR China; b Key Laboratory of Plant Secondary Metabolism and Regulation of Zhejiang Province, College of Life Sciences and Medicine, Zhejiang Sci-Tech University, Hangzhou, PR China

**Keywords:** Brassicaceae, chloroplast genome, *Neomartinella yungshunensis*, phylogenetic analyses

## Abstract

*Neomartinella yungshunensis* (W. T. Wang) Al-Shehbaz 2000 is a kind of perennial herb usually distributed in Yongshun County, Xiangxi Tujia Miao Autonomous Prefecture, Hunan Province. It was the first time to report the complete chloroplast genome sequence of *N. yungshunensis*. The complete chloroplast genome was 152,597 bp in size, including a large single-copy (LSC) region of 83,145 bp, a small single copy region (SSC) of 17,400 bp, and a pair of reverse repeats (IR) of 26,026 bp. It contained 133 genes in the chloroplast genome, including 87 protein-coding genes (PCGs), 37 transfer RNA (tRNA) genes, and 8 ribosomal RNA (rRNA) genes. The GC content of the chloroplast genome was 36.4%. The phylogenetic analysis showed that *N. yungshunensis* is closely related to *Eutrema integrifolium* (NC_049636).

*Neomartinella yungshunensis* (W. T. Wang) Al-Shehbaz is a kind of perennial herb that played an important role in Chinese culture, an uncommonly found wild plant, which first reported in Yongshun County, Xiangxi Tujia Miao Autonomous Prefecture, Hunan Province. To better understand the relationships among *N. yungshunensis* and other Cruciferous species, whole chloroplasts were sequenced and analyzed within a phylogenetic context.

In this study, the fresh leaves tissue of *N. yungshunensis* were collected from a mountain with an altitude of 455 m, in Yundou town, Shiquan County, Ankang City, Shaanxi Province (32°47′53″N, 108°8′8″E). The species identified by Lulu Xun who was from Xi’an Botanical Garden. The voucher specimen was preserved at XBGH (The Herbarium of Xi’an Botanical Garden, http://www.xazwy.com) (Voucher number: *Lulu Xun* et al SQ012 and xunlulu@xab.ac.cn). Total genomic DNA was extracted using the modified CTAB method (Doyle 1987). The extracted DNA was deposited at Key Laboratory of Plant Secondary Metabolism and Regulation of Zhejiang Province, Zhejiang Sci-Tech University (http://sky.zstu.edu.cn) under the voucher number ZSTUX0018 (collected by Pengguo Xia and xpg_xpg@zstu.edu.cn). With the aid of Illumina Hiseq X Ten sequencer, the genomic library for Illumina paired-end (PE) sequencing was constructed. Then, the software NOVOPlasty version 2.7.2 (Dierckxsens et al. 2017) was used to assemble the complete chloroplast genome of *N. yungshunensis*.

We annotated the chloroplast genome using Geneious Prime software, and the chloroplast genome of *Eutrema integrifolium* (GenBank accession number NC_049636) was cited as a reference annotation. The chloroplast genome annotation of *N. yungshunensis* was thus obtained and submitted to Genbank (GenBank accession number MW981639). The complete chloroplast genome sequence of *N. yungshunensis* was 152,597 bp in length, including a large single copy (LSC) region of 83,145 bp, a small single copy region (SSC) of 17,400 bp, and a pair of reverse repeats (IR) of 26,026 bp. The overall GC content of the whole plastid genome was 36.4%. The genome contained 133 genes, including 37 transfer RNA (tRNA) genes, 8 ribosomal RNA (rRNA) genes, and 87 protein-coding genes (PCGs).

A phylogenetic tree was constructed based on the complete chloroplast genomes of *N. yungshunensis* and related species, including other species of the Cruciferae. The maximum likelihood (ML) was generated by the software IQTREE version 1.6.7 (Nguyen et al. 2015) with the best selected TVM + F +R2 model and 1000 bootstrap repeats. From the phylogenetic tree, it is not difficult to see that *N. yungshunensis* has the closest relationship with *Eutrema integrifolium* (GenBank accession number NC_049636) had the closest relationship. Furthermore, the phylogenetic tree also reflected the relationship among *N. yungshunensis* and other species (Figure 1). The complete chloroplast genome sequence of *N. yungshunensis* can provide necessary data for phylogenetic studies of Brassicaceae. It is hoped that this study will help resolve the intrageneric and interspecific phylogeny of Cruciferae.

**Figure 1.**
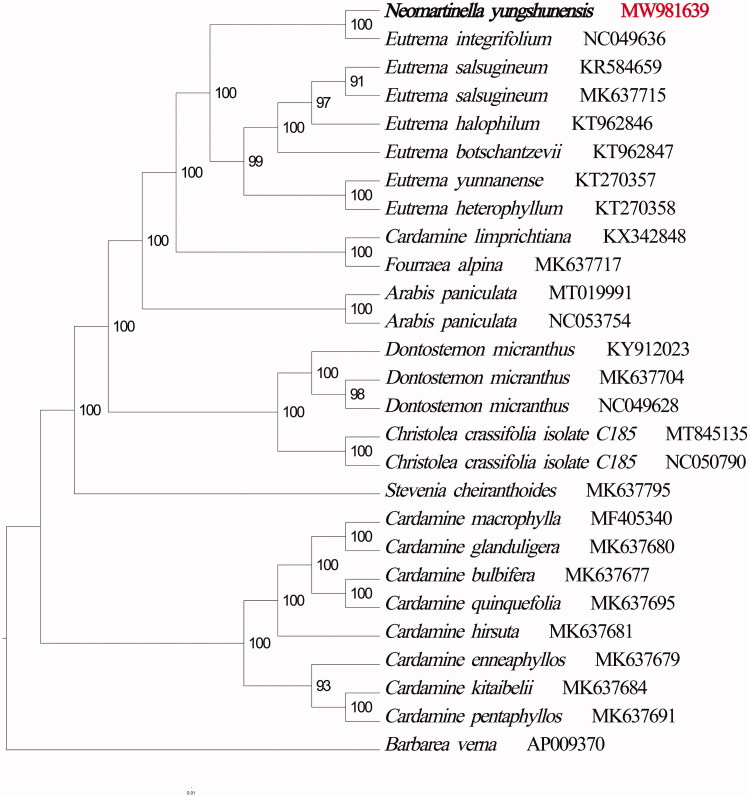
A phylogenetic tree of *Neomartinella yungshunensis*, 25 related species and an outgroup was constructed based on the complete chloroplast genome using the maximum likelihood (ML) method. Numbers in each node indicated the bootstrap support values.

## Ethical approval

Research and collection of plant material was conducted according to the guidelines provided by Xi’an Botanical Garden. Permission was granted by Hangzhou Academy of Agricultural Sciences to carry out research on the species.

## Author contributions

W.C., J.Q and P.X. conceived and designed this study. X. C. and H. L. conducted analysis. J.Q, P.X. and X.C. contributed the analytical methods. W.C. wrote the manuscript. P.X. edited the manuscript. All authors have read and agreed to the published version of the manuscript.

## Data Availability

The data that support the findings of this study are openly available in NCBI (https://www.ncbi.nlm.nih.gov) GenBank with the accession number (MW981639). The associated BioProject, SRA, and BioSample numbers of the funding are PRJNA723133, SRR14278903, and SAMN18805689 respectively.
